# A study of TiO_2_/carbon black composition as counter electrode materials for dye-sensitized solar cells

**DOI:** 10.1186/1556-276X-8-227

**Published:** 2013-05-14

**Authors:** Jeongmin Lim, Sang Yeoul Ryu, Jeonghun Kim, Yongseok Jun

**Affiliations:** 1 Interdisciplinary School of Green Energy, Ulsan National Institute of Science and Technology (UNIST), Ulsan 689-798, Republic of Korea; 2 Fine Chemical and Material Technical Institute, Ulsan Techno Park, Ulsan 681-802, Republic of Korea

**Keywords:** Dye-sensitized solar cells, Carbon black, Counter electrode, Nano composite

## Abstract

This study describes a systematic approach of TiO_2_/carbon black nanoparticles with respect to the loading amount in order to optimize the catalytic ability of triiodide reduction for dye-sensitized solar cells. In particular, the cell using an optimized TiO_2_ and carbon black electrode presents an energy conversion efficiency of 7.4% with a 5:1 ratio of a 40-nm TiO_2_ to carbon black. Based on the electrochemical analysis, the charge-transfer resistance of the carbon counter electrode changed based on the carbon black powder content. Electrochemical impedance spectroscopy and cyclic voltammetry study show lower resistance compared to the Pt counter electrode. The obtained nanostructures and photo electrochemical study were characterized.

## Background

Dye-sensitized solar cells (DSSCs) have attracted considerable attention as a viable alternative to conventional silicon-based photovoltaic cells [1] because of their low-production cost, high conversion efficiency, environmental friendliness, and easy fabrication procedure [2-5]. A typical DSSC is comprised of a nanocrystalline semiconductor (TiO_2_), an electrolyte with redox couple (I_3_^−^/I^−^), and a counter electrode (CE) to collect the electrons and catalyze the redox couple regeneration [6]. Extensive researches have been conducted in order for each component to achieve highly efficient DSSCs with a modified TiO_2_[7], alternative materials [8,9], and various structures [10-12]. Usually, Pt-coated fluorine-doped tin oxide (FTO) is used as a counter electrode owing to its superior catalytic activity [13]. However, there are researches reporting that Pt corrodes in an electrolyte containing iodide to generate PtI_4_[14,15]. Besides, large solar module systems will benefit from materials that are abundantly available with high chemical stability. Therefore, it is necessary to develop alternative materials which must be inert and show good catalytic effect in the electrolyte.

A great deal of effort has been taken to replace the Pt metal with other materials such as cobalt sulfide (CoS) [16], titanium nitrides (TiN) [17-19], and carbon derivatives [20-23]. Among these candidates, carbon materials obtain increasing attention due to their abundance, low cost, and high catalytic activities with chemical stability against iodine redox couples [24-27].

Here, we focus on carbon black which is produced by combustion of heavy petroleum products with high surface areas. Compared to any other forms of carbon derivatives, carbon black does not require a delicate process to apply to counter electrodes. Note that carbon nanotubes and nanorods require multiple operations for the synthesis and application on counter electrode substrates. In this work, we demonstrate the properties of carbon black material with anatase TiO_2_ in an attempt to replace the Pt counter electrode in DSSC applications. Forty-nanometer-sized TiO_2_ nanoparticles were tested with various weight ratios of carbon black, and the effect was investigated by electrochemical impedance spectroscopy and cyclic voltammetry analysis in detail.

## Methods

### Carbon black

The carbon black chunk was purchased from Sigma-Aldrich (14029-U, St. Louis, MO, USA) and ground to make powder. Pulverized carbon black was sifted out with 80-unit mesh then calcined for 2 h at 500°C in a muffle furnace. The annealed carbon mass was ground again and passed through with 200- to 350-unit mesh for further heat treatment at 300°C for 2 h in order to remove the impurities. The final carbon black powder size was 80 nm.

### Anatase TiO_2_ nanocrystal synthesis

Titanium dioxide nanoparticles in anatase crystal form were synthesized by a modified Burnside method [28]. A 162-mL titanium (IV) isopropoxide (0.5 M, Sigma-Aldrich) was rapidly injected into 290 mL of distilled water (15.5 mol, J. T Baker, Avantor Performance Materials, Center Valley, PA, USA) under stirring, and the solution was vigorously stirred for a further 10 h. Addition of titanium (IV) isopropoxide in such an aqueous solution results in a white precipitate in the TiOx form. The resultant colloid was filtered and washed thrice with 50 mL of deionized (DI) water. Then the filtrate was loaded into an autoclave with 30 mL of a 0.6 M tetramethylammonium hydroxide solution to form a white slurry. The pH of the colloidal solution after addition of the base was measured to be between 7 to approximately 8. The solution was heated to 120°C for 6 h in order to obtain a peptization, and then the peptized suspension was treated hydrothermally in the autoclave at a temperature of 200°C for 4.5 h. The colloids were centrifuged at 13,000 rpm for 40 min and the precipitate was dried for 1 day in a vacuum oven, then dissolved into the DI water (wt.% of DI water/TiO_2_ = 20:1). Then, a clear white color precipitate was observed.

### TiO_2_/carbon black slurry preparation

The TiO_2_ and carbon black (T/CB) slurry was prepared as follows: various amounts of carbon black powder (50, 100, 200, and 500 mg) were mixed with 40-nm sizes of TiO_2_ nanoparticles in various weight ratios (T/CB; 10:1, 5:1, 2.5:1, and 1:1). The mixture was dispersed by ultrasonication (750 W, Sonics & Materials, Inc, Newtown, CT, USA) for 10 min. After the ultrasonic treatment, 100 μl of Triton X-100 (Sigma-Aldrich) was added to the mixture and further ultrasonic treatment was carried for 10 min.

### Electrodes and cell fabrication

Samples of fluorine-doped tin oxide substrate (Pilkington TEC Glass-TEC 8, Nippon Sheet Glass Co., Ltd, Tokyo, Japan) were washed in a detergent solution, DI water, an ethanol-acetone mixture solution (*v/v* = 1/1), and 2-propanol in an ultrasonic bath for 5 min, in turn, and then treated by a UV-O_3_ system for 15 min to introduce a hydrophilic surface. Nanocrystalline TiO_2_ paste (20 nm, ENB-Korea, Daejeon, Korea) was coated onto the FTO glasses using a doctor blade. The TiO_2_-coated FTO glasses were annealed at 500°C for 1.5 h to create a TiO_2_ film; then, the substrate was treated with 40 mM of an aqueous solution of TiCl_4_ at 80°C for 30 min and rinsed with DI water and an ethanol-acetonitrile mixture solution (*v/v* = 1/1). The substrate was heat-treated again at 500°C for 30 min and immersed in 0.3 mM (Bu_4_N)_2_[Ru(dcbpyH)_2_(NCS)_2_] (N719) in a mixed solvent of acetonitrile and *tert*-butanol (*v/v* = 1/1) with 0.075 mM DINHOP for 24 h. To prepare counter electrodes, a 10-M H_2_PtCl_6_ solution in ethanol and T/CB slurry of various weight ratios were coated onto a cleaned FTO glass separately, followed by annealing at 500°C for 1 h in a tube furnace. The working electrode and the counter electrode were sandwiched together using a 50-μm thick Surlyn (DuPont) at 100°C for 10 s. An electrolyte containing a mixture of 0.6 M 1-hexyl-2,3-dimethyl-imidazolium iodide, 0.1 M guanidine thiocyanate, 0.03 M iodine, and 0.5 M 4-*tert*-butylpyridine in acetonitrile was injected, and final sealing completed the fabrication of the cell.

## Results and discussion

**Figure 1:**
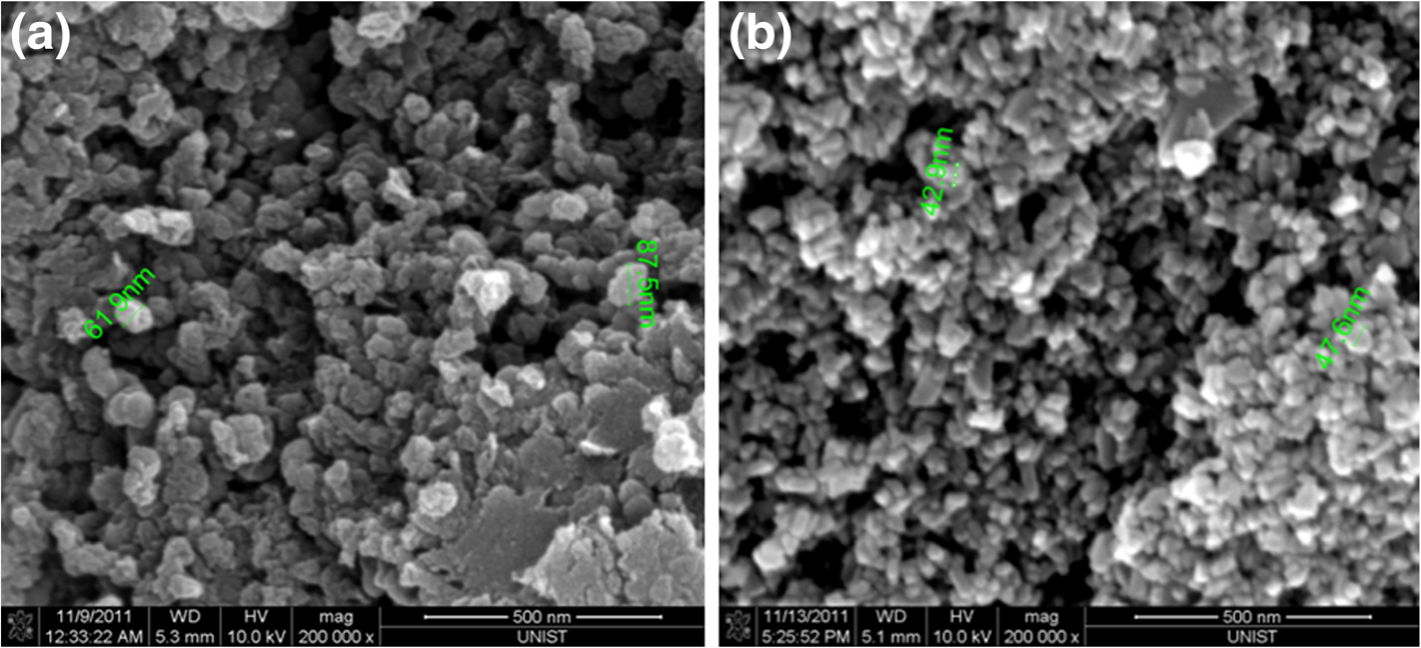
**FE-SEM image of the (a) carbon black powder and (b) hydrothermally synthesized TiO**_**2 **_**nanoparticles.**

Figure 1 shows surface morphologies of the pure carbon black and the synthesized TiO_2_ nanoparticles. The sizes of carbon black and TiO_2_ particles are 75 and 40 nm, respectively. The carbon black has a lot of active sites for catalysis at edges with high porosity at approximately 75-nm size, and TiO_2_ can easily be attached onto the FTO substrate at 40-nm size. We applied the mixture of both nanoparticles as a counter electrode; pores for electron transfer with high surface area and good adhesion of catalytic materials can easily be made.

**Figure 2:**
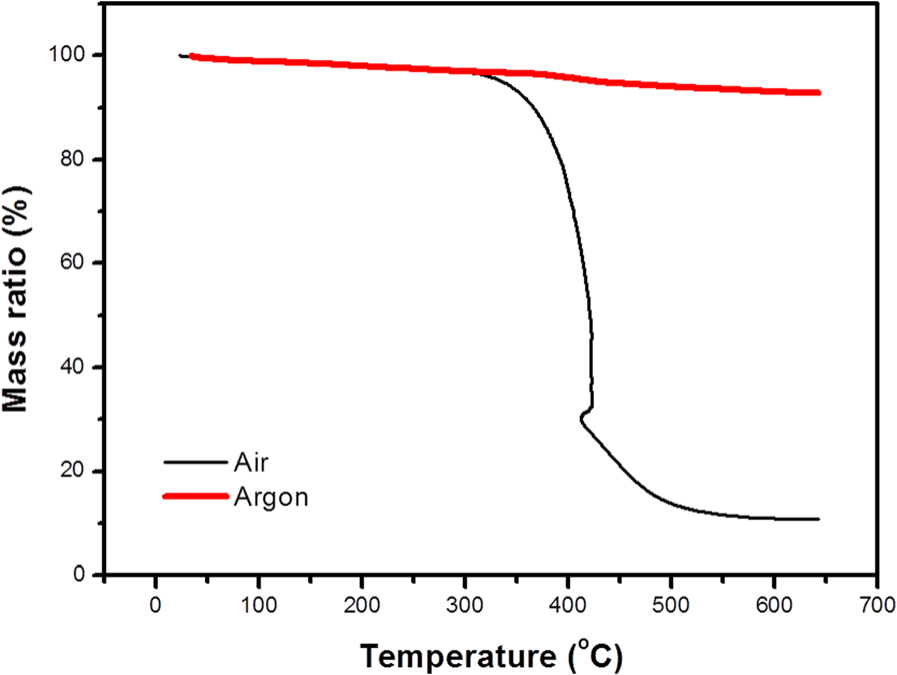
TGA in air and argon with the carbon black at a heating rate of 10°C/min.

Figure 2 shows a thermogravimetric analysis (TGA) of carbon black under air and argon atmosphere. When it reaches 350°C, TGA data show a very similar decrease for both conditions, which indicates that any organic residue on the surface evaporates. However, the carbon black in air showed drastic weight loss starting at approximately 350°C, possibly due to combustion. No noticeable decrease in weight is observed in the argon atmosphere sample until approximately 650°C. To avoid degradation, an argon atmosphere was used and the temperature of calcination was set at 500°C to remove all residues in the carbon black and improve the contact of TiO_2_.

**Figure 3:**
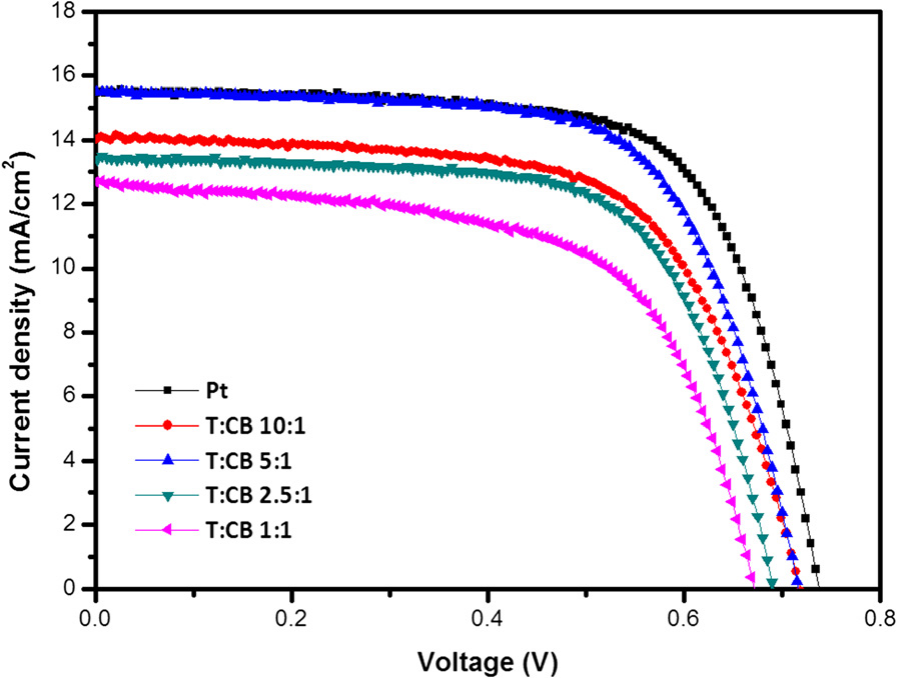
Photocurrent-voltage curves of the devices.

The ratios of T/CB slurry were varied from 10:1, 5:1, and 2.5:1 and 1:1 weight ratio for the counter electrode. *J*-*V* curves for each ratio of T/CB slurry are shown in Figure 3, and the performance of these cells is listed in Table 1. The reference Pt cell shows 7.7% efficiency (*η)* with a 69.3% fill factor (*FF*), and the 5:1 ratio sample shows similar efficiency (7.4%) with a comparable *FF* (67.4%) and short-circuit current (*J*_sc_) (15.5 mA/cm^2^). Other samples show similar open-circuit potential (*V*_oc_) and *FF*, but the *J*_sc_ are much lower than the Pt or 5:1 ratio cases. When the amount of carbon black is low (10:1 ratio), the adhesion of T/CB slurry to the FTO is better. However, reduction of I_3_^−^ is not active due to the low surface area available for triiodide reduction and it shows slightly lower *J*_sc_ than the 5:1 ratio sample. A large amount of carbon black (2.5:1, 1:1 ratios) has enough surface area of reduction, but the poor adhesion of FTO and carbon black makes it difficult to get high efficiency [15,27,29].

**Table 1:** **Photovoltaic performance of Pt and TiO**_**2**_**/carbon black composites as counter electrode**

**Composite**	***J***_**sc **_**(mA/cm**^**2**^**)**	***V***_**oc **_**(V)**	***FF *(%)**	***η (%)***
Pt	15.5	0.73	69.3	7.7
T/CB (10:1)	14.1	0.71	64.6	6.6
T/CB (5:1)	15.5	0.71	67.4	7.4
T/CB (2.5:1)	13.5	0.69	68.7	6.5
T/CB (1:1)	12.6	0.66	61.3	5.1

**Figure 4:**
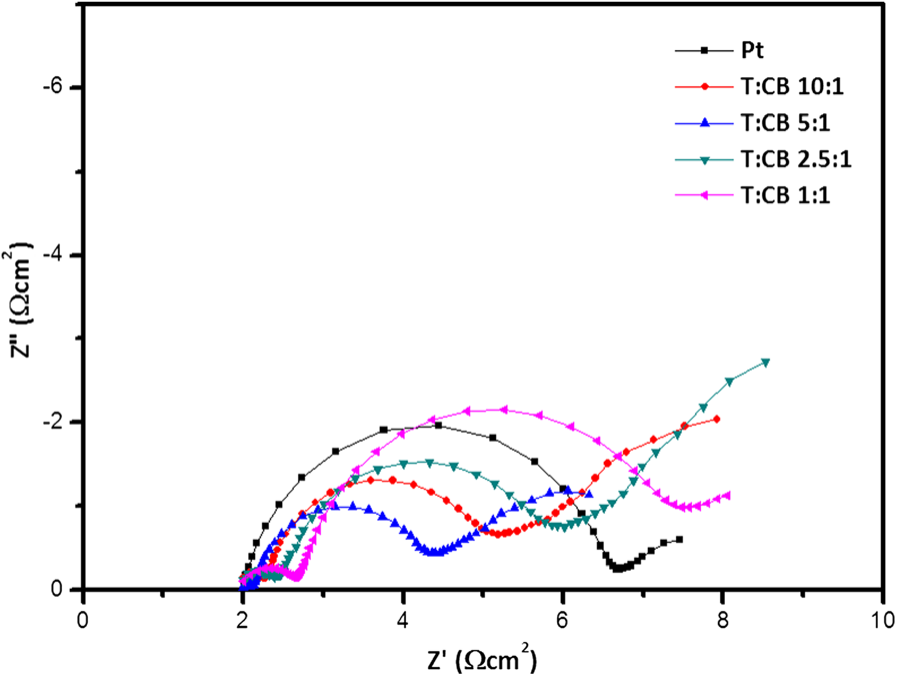
Nyquist plot of Pt reference cell and four different ratios of T/CB symmetrical cells.

Electrochemical impedance spectroscopies (EIS) of a dummy cell were analyzed to determine the interfacial electrochemical properties with ratios of T/CB. Figure 4 shows the Nyquist plots of symmetric cells with T/CB slurry ratios of 10:1, 5:1, 2.5:1, and 1:1 and a conventional Pt-coated counter electrode. The first arc of the Pt-based counter electrodes appears at 100,000 to approximately 100 Hz with only one spectrum of Pt electrode/electrolyte interface. Under 100 Hz, Warburg was obtained by electrolyte diffusion in the dummy cell. For the T/CB counter electrodes, impedance spectra exhibit three separated semicircles, which correspond to resistances at the counter electrode/electrolyte interface *R*_ct_, the TiO_2_/carbon black interface, and the electrolyte diffusion Zw [30]. The *R*_ct_ value is directly related to the amount of carbon content in turn of the number of catalytic sites. The higher amount of carbon content should lead to the lower *R*_ct_ value. It has been observed that the *R*_ct_ value of T/CB = 5:1 composite is lower than the T/CB = 10:1 due to the higher amount of carbon content which provides more catalytic sites for the reduction reaction. The composites T/CB = 2.5:1 and T/CB = 1:1 have even more amount of carbon content than the other two composites (T/CB = 10:1 and T/CB = 5:1 ratios), the former set showed higher *R*_ct_ value than the later set due to their poor interconnection between T and CB as well as the poor adherence property with the FTO surface. The low frequency semicircle has a similar shape for all the T/CB composite cells because the diffusion in the electrolyte is invariant with the catalytic activity of the electrodes.

**Figure 5:**
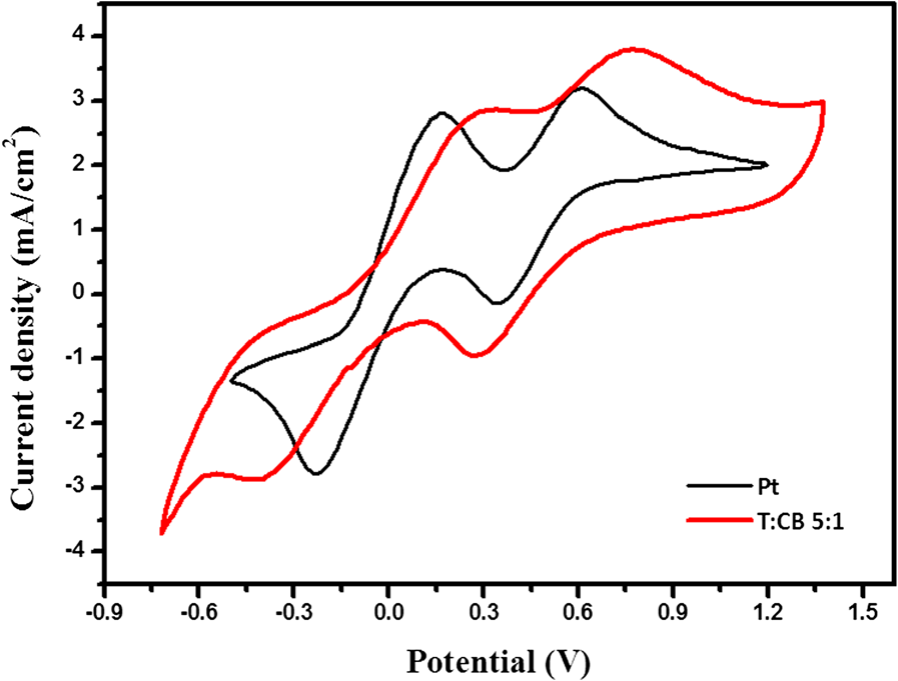
Cyclic voltammograms of Pt reference cell and optimized T/CB cell.

To further elucidate the electrochemical properties, the samples with the best-performing counter electrode were investigated by a cyclic voltammetry (CV) test with a scan rate of 50 mV/s. As shown in Figure 5, the counter electrodes based on the best-performing T/CB composites and Pt show similar shapes in terms of redox peak position with increased current density. In the CV curves, two pairs of redox peaks were obtained. The positive side, known as anodic, refers to the oxidation of iodide and triiodide, and the negative (cathodic) side refers to the reduction of triiodide. The reduction/oxidation peaks for the Pt and the T/CB composites are shown at −0.224 V/0.163 V and −0.394 V/0.333 V, respectively. The shift might be due to the higher *R*_ct_ between carbon black and the electrolyte. However, the T/CB composites exhibited comparable current density with the Pt electrode, and it indicates that the T/CB composites have higher intrinsic catalytic activity for redox reaction of iodide ions.

Finally, it should be noted that a key advance in this study is the integration of high-quality DSSC counter electrode device design for the reduction of triiodide in the DSSC system. CV, EIS, and photocurrent-voltage analysis consistently confirm the excellent catalytic activities of the synthesized and optimized TiO_2_/carbon black composites, which are comparable to that of the Pt counter electrode. The prepared counter electrode effectively utilized the reduction of triiodide to iodide. In this architecture, the influence of various amounts of carbon black and TiO_2_ loading can be explained. To get the high percolation of electrolyte and high surface area of catalytic sites, 40-nm TiO_2_ nanoparticles were applied as a binder of carbon black and at the ratio of 5:1, T/CB shows comparable efficiency with Pt electrode.

## Conclusion

In summary, composites made of carbon black with 40-nm TiO_2_ nanoparticles have been synthesized using the hydrothermal method. Different weight ratios of carbon black containing TiO_2_ composites have been tested as the counter electrode material in order to analyze the catalytic performance of triiodide reduction reaction. The best optimized condition at a 5:1 ratio of TiO_2_ and carbon black showed the overall efficiency of 7.4% while the well-known Pt as the counter electrode at the same condition shows 7.7% efficiency. The fill factors were strongly dependent on the loading of the carbon black powder and found to be around 68%. Interfacial charge transfer and mass transport were characterized by cyclic voltammetry and electrochemical impedance spectroscopy. This technique of synthesizing nanostructures for high surface area along with optimum carbon black loading afforded an effective and simple way to replace the Pt-based counter electrode for DSSC. Overall, the TiO_2_/carbon black-based DSSC showed excellent cell efficiency that rivals cells with a Pt-based CE and exhibited remarkable electrocatalytic activity. This work provides an intriguing way of structurally designing a low-cost, Pt-free, high-performance CE material for DSSCs.

## Competing interests

The authors declare that they have no competing interests.

## Authors’ contributions

JL participated in the design of the study, carried out the experiments, and drafted the manuscript. SYR and JK carried out the sample preparation and measurements. YJ supervised the work. All authors read and approved the final manuscript.
